# Adenoids and Trachoma

**Published:** 1903-01-31

**Authors:** 


### ADENOIDS AND TRACHOMA.

At a recent meeting of the New York Academy of Medicine Dr. Ralph Opdyke[1] read a paper in which he drew attention to the marked relationship existing between trachoma and adenoids, a subject which had been forcibly brought to his notice as the result of examinations made on the children of the city at the request of the Commissioner of Health. The children were examined with regard especially to catarrhal and contagious diseases of the eyes. In the course of these examinations of many thousands of children adenoid vegetations of the superior and posterior naso-pharyngeal space were so frequently met with in cases affected with severe trachoma that he was forced to take into serious consideration the relationship of these two diseases. Especially was this the case in the so-called "operative" forms of the disease where on an average two out of every three cases had pronounced adenoid vegetations with all the typically marked symptoms of this affection. The same condition was found to be present not only in the lower East Side schools, but also in the uptown West Side schools, notwithstanding their better facilities and healthier children. The same connection has also been observed among the trachoma cases assigned to Dr. Opdyke for treatment at the various hospitals with which he is connected. To prove that the abnormally large adenoid vegetation in the pharynx is an antecedent to the trachoma complication of the lid, we need but recall that the adenoid vegetation is present at birth, while the lymphoid structure in the lid is not sufficiently developed for the inception of the insidious trachoma until the child is several years old. In regard to the causes which lead to the production of trachoma, little as may be positively known, all authorities seem to be agreed upon one fact—namely, that the liability to attacks depends most strongly upon the state of the general health at the time of exposure to the infection; in other words, that there must needs be a condition of lowered vitality due to errors of diet, hygiene, environment, etc.; and Dr. Opdyke claims that none of these errors can bring about the required condition of lowered vitality more forcibly than the evil results which arise from the presence of adenoid vegetations in the pharynx. Hence he holds that to prevent the trachoma we ought to remove the adenoids.

[1] New York Med. Rec., Jan. 3.

